# MicroRNA-100 and microRNA-21 as markers of survival and chemotherapy response in pancreatic ductal adenocarcinoma UICC stage II

**DOI:** 10.1186/s13148-015-0166-1

**Published:** 2015-12-23

**Authors:** Sameer Abdallah Dhayat, Baha Abdeen, Gabriele Köhler, Norbert Senninger, Jörg Haier, Wolf Arif Mardin

**Affiliations:** Department of General and Visceral Surgery, University Hospital Muenster, Albert-Schweitzer-Campus 1 (W1), 48149 Muenster, Germany; Department of Pathology, University Hospital Muenster, Albert-Schweitzer-Campus 1 (D17), 48149 Muenster, Germany; Comprehensive Cancer Center Muenster, University Hospital Muenster, Albert-Schweitzer-Campus 1 (W1), 48149 Muenster, Germany

**Keywords:** Pancreatic ductal adenocarcinoma, Chemoresistance, microRNA

## Abstract

**Background:**

Pancreatic ductal adenocarcinoma (PDAC) remains a highly chemoresistant tumor entity for which no reliable molecular targets exist to predict or influence the success of chemotherapy. Recently, we identified a panel of microRNAs associated with induced gemcitabine chemoresistance in human PDAC cell lines. This clinical study evaluates these microRNAs and associated molecular markers as prognostic markers of outcome in 98 PDAC patients Union Internationale Contre le Cancer (UICC) stage II undergoing curative surgery with adjuvant gemcitabine chemotherapy. The primary end points of this study are recurrence-free survival and overall survival.

**Results:**

Poor response to chemotherapy was significantly correlated to overexpression of microRNA-21 (*p* = 0.029), microRNA-99a (*p* = 0.037), microRNA-100 (*p* = 0.028), and microRNA-210 (*p* = 0.021) in tissue samples of PDAC patients UICC stage II. Upregulation of these microRNAs was associated with a significantly shorter overall survival and recurrence-free survival (*p* < 0.05). Overexpression of phosphatase and tensin homolog (PTEN) (*p* = 0.039) and low expression of multidrug resistance (MDR)-1 (*p* = 0.043) and breast cancer resistance protein (BCRP)-1 (*p* = 0.038) were significantly correlated to improved response to adjuvant chemotherapy. Adjuvant gemcitabine treatment (*p* < 0.0001) and low tumor grading (*p* = 0.047) were correlated to better outcome. MicroRNA-100, microRNA-21, and its targets PTEN and MDR-1 were independent factors of survival in multivariate analysis.

**Conclusions:**

Multivariate survival analyses identified microRNA-21 and microRNA-100 as unfavorable prognostic factors in resected and adjuvant treated PDAC UICC stage II patients.

## Background

Pancreatic ductal adenocarcinoma (PDAC) remains one of the most deadly cancers in western countries with a median survival below 6 months and a dismal 5-year overall survival rate of less than 5 % [1]. Despite developments in novel diagnostic and surgical techniques over the past decades, only morbidity and postoperative mortality were improved, without significant impact on survival. Lack of early symptoms combined with rapid disease progression result in low resectability rates of about 15 % of PDAC patients with 5-year survival rates below 20 % [2].

Adjuvant first-line chemotherapy and palliative treatment by gemcitabine have slightly improved the clinical outcome. However, nearly half of the treated patients do not benefit from gemcitabine-based therapies [3, 4]. Chemotherapy response markers are needed to identify PDAC patients who may benefit from adjuvant gemcitabine chemotherapy.

PDAC chemoresistance is acquired through multiple molecular pathways and genetic alterations affecting cell cycle, apoptosis, and intracellular drug accumulation. Especially, members of the ATP binding cassette (ABC) superfamily including multidrug resistance (MDR)-1 P-glycoprotein, multidrug resistance protein (MRP)-1, and breast cancer resistance protein (BCRP)-1 have been shown to mediate resistance against several anticancer drugs by enhanced drug efflux across cellular plasma membranes [5]. Deoxycytidine kinase (DCK), a key activator of gemcitabine, and its regulator Hu antigen R (HuR) have been identified as powerful independent prognostic factors for PDAC patients undergoing adjuvant gemcitabine therapy [6–8]. Further, mutations and epigenetic downregulation of phosphatase and tensin homolog (PTEN), a tumor suppressor and the most important negative regulator of Akt/PI3K signaling, are regarded as crucial for PDAC development and chemoresistance [9].

Although a number of molecular players have been connected to chemoresistance, their clinical impact remains controversial [10].

We previously identified a panel of microRNAs associated with induced gemcitabine chemoresistance in human PDAC cell lines: Two PDAC cell lines were treated by repeated pulsatile gemcitabine treatment to induce acquired chemoresistance. Global microRNA profiling identified a panel of 16 significantly and concordantly dysregulated microRNAs in both chemoresistant PDAC cell clones [11].

In this clinical study, our aim was to validate these microRNA candidates as prognostic markers of chemotherapy response to gemcitabine in patients with resected PDAC stage II according to the Union Internationale Contre le Cancer (UICC).

## Results

### Clinicopathologic factors and chemotherapy response as survival markers

A total of 91 PDAC patients UICC stage II with curative pancreatic head (82.4 %) or left resection (17.6 %) and 13 patients with benign, noninflammatory pathology were analyzed (Table 1). Seven of initially 98 PDAC patients with an in-hospital-mortality of ≤30 days were excluded. The median age at diagnosis was 66 years, and the male to female ratio was approximately 1.2:1. The distribution by stage was 31.9 % UICC stage IIa and 68.2 % Stage IIb. The majority of patients (65; 71.4 %) had died by the time of final analysis with a median follow-up time of 30.5 months for the 26 patients still alive. The median survival time for all patients was 22 months.

**Table 1:** Clinicopathologic characteristics of PDAC stage II patients

	patients *n*	Median overall survival (months)	95 % CI	*p*
Total	91	21	16–26	
Age (years)				
≤65	41	27	21–33	0.1754
>65	50	19	16–22	
Gender				
Female	41	26	18–34	0.8796
Male	50	20	15–25	
UICC stage				
IIa	29	27	13–40	0.3347
IIb	62	20	16–24	
Grading				
G1 and G2	55	26	1–31	0.0474
G3	36	15	8–22	
Perineural invasion				
Pn0	32	19	9–29	0.7835
Pn1	59	21	16–26	
Vene invasion				
V0	74	23	16–30	0.5583
V1	17	21	17–25	
Lymphatic invasion				
L0	50	24	18–29	0.9821
L1	41	21	15–26	
Resection margin				
R0	77	23	15–31	0.3653
R1	24	18	16–26	
Type of surgery				
Pancreatic head resection	75	32	17–25	0.2475
Traverso-Longmire	66			
Whipple-Kausch	9			
Pancreatic left resection	16	21	14–49	
Adjuvant gemcitabine				
Yes	64	31	24–37	2.9922E-8
No	27	12	9–14	

Indication of median overall survival in months and 95 % confidence interval (CI). *p* < 0.05 indicates significance

**Fig. 1:**
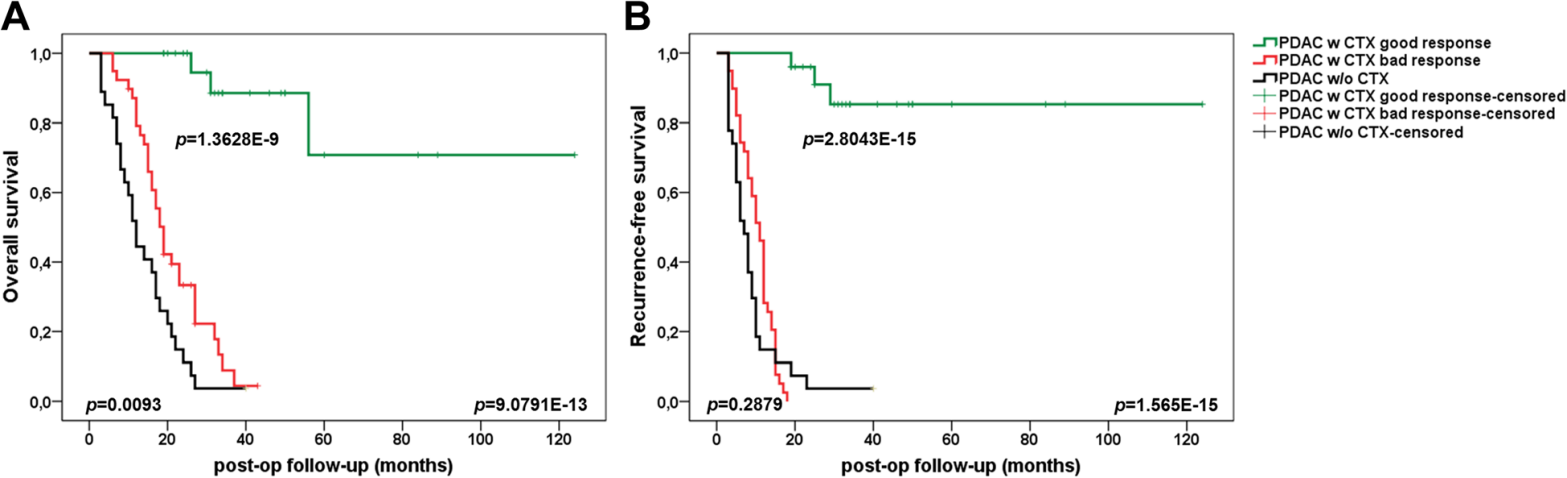
Prognostic impact of adjuvant gemcitabine treatment in PDAC UICC stage II patients. Improved overall (**a**) and recurrence-free survival (**b**) in patients with adjuvant gemcitabine treatment and good (*n* = 25) vs. bad (*n* = 39) response (*p* = 1.3628E-09; *p* = 2.8043E-15) and vs. no adjuvant gemcitabine treatment (*n* = 27) (*p* = 9.0791E-13; *p* = 1.565E-15). Improved overall survival in patients with bad chemotherapy response vs. no adjuvant treatment (*p* = 0.0093)

Univariate analyses indicated that tumor grading (*p* = 0.0474) correlated with worse overall survival. Patients who underwent adjuvant gemcitabine chemotherapy had significantly improved overall survival rates (*p* = 2.9922E-8) (Table 1). Patients with adjuvant gemcitabine treatment and good response (determined by median split) showed improved median overall and recurrence-free survival vs. bad response (33 months, 95 % confidence interval (CI) 26–49 vs. 19 months, 95 % CI 15–13, *p* = 1.3628E-09 and 32 months, 95 % CI 25–46 vs. 11 months, 95 % CI 8–12, *p* = 2.8043E-15) and vs. no adjuvant treatment (12 months, 95 % CI 8–17, *p* = 9.0791E-13 and 7 months, 95 % CI 5–9, *p* = 1.565E-15). Improved overall survival was even shown in patients with poor chemotherapy response vs. no adjuvant treatment (*p* = 0.0093) (Fig. 1). Subgroup analysis of response to chemotherapy groups (group II vs. III) revealed no significant differences in the distribution of clinicopathologic factors (Table 2).

**Table 2:** Clinicopathologic characteristics of PDAC patients UICC stage II with different adjuvant chemotherapy response

	Total	PDAC II w/o CTX	PDAC II w CTX		*P*	*P*
			Good response	Bad response		
	(*n* = 91)	I (*n* = 27)	II (*n* = 25)	III (*n* = 39)		II vs. III
Age (years)						
≤65	41	10	12	19	0.5092	1
median (range)		63 (54–65)	56 (41–64)	53 (31–65)		
>65	50	17	13	20		
median (range)		72 (66–84)	73 (66–84)	71 (67–79)		
Gender						
Female	41	14	11	16	0.6801	1
Male	50	13	14	23		
UICC stage						
IIa	29	7	9	13	0.6469	1
IIb	62	20	16	23		
Grading						
G1	5	0	3	2	0.0231	0.5008
G2	50	10	16	24		
G3	36	17	6	13		
Perineural invasion						
Pn0	32	10	10	12	0.7305	0.5904
Pn1	59	17	15	27		
Vene invasion						
V0	74	23	22	29	0.3256	0.2204
V1	17	4	3	10		
Lymphatic invasion						
L0	50	17	15	18	0.3367	0.3149
L1	41	10	10	21		
Resection margin						
R0	67	20	21	26	0.3071	0.1553
R1	24	7	4	13		
Type of surgery						
Pancreatic head resection	75	26	17	32	0.0276	0.2347
Traverso-Longmire	66	22	15	29		
Whipple-Kausch	9	4	2	3		
Pancreatic left resection	16	1	8	7		
Adjuvant gemcitabine						
Yes	64	0	25	39	0.0000	1
No	27	27	0	0		
Tumor recurrence						
≤18 months	63	24	0	39	0.0000	0.0001
>18 months	5	2	3	0		
Tumor related death						
≤18 months	39	20	0	19	0.0234	0.0856
>18 months	22	6	3	13		

*p* < 0.05 indicates significance

### MicroRNAs as markers of chemotherapy response and survival

**Fig. 2:**
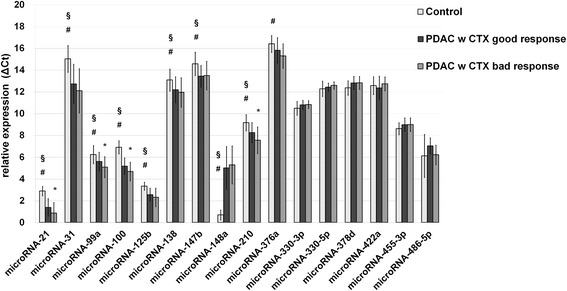
ΔCT expression level (means with standard deviation) of in vitro chemoresistant-specific microRNAs in benign and cancerous human pancreatic specimen. ΔCt levels are inversely proportional to the amount of target microRNA in the sample. *Asterisk* indicates to a significant difference of *p* < 0.05 between PDAC patients with good and poor chemotherapy (w CTX) response. *Number sign* and *section sign* indicate to a significant difference of *p* < 0.05 between benign noninflammatory pancreas controls and PDAC with good or bad CTX response, respectively

Macrodissected PDAC tissue of patients with poor (*n* = 39) and good chemotherapy response (*n* = 25) was evaluated by quantitative real-time PCR (qRT‐PCR) for the expression of in vitro chemoresistance-specific microRNAs (Fig. 2, Table 3). MicroRNA-21 (*p* = 4.3817E-07), microRNA-31 (*p* = 0.0003), microRNA-99a (*p* = 0.0406), microRNA-100 (*p* = 4.0492E-08), microRNA-125b (*p* = 0.0001), microRNA-138 (*p* = 0.0301), microRNA-147b (*p* = 0.0028), and microRNA-210 expression (*p* = 0.0044) were significantly upregulated in PDAC stage II vs. benign controls. Further, microRNA-148 was significantly downregulated in PDAC tissues (*p* = 3.9814E-09).

**Table 3:** MicroRNA expression of PDAC patients with good and poor chemotherapy (w CTX) response

	Control vs. PDAC w CTX good response	Control vs. PDAC w CTX bad response	PDAC w CTX good response vs. PDAC w CTX bad response
microRNA-21	2.89 vs. 1.39 (+51.6 %) *p* = 4.3817E-07	2.89 vs. 0.86 (+70.3 %) *p* = 3.3364E-09	1.39 vs. 0.86 (+38.7 %) *p* = 0.0295
microRNA-31*	15.02 vs. 12.74 (+15.2 %) *p* = 0.0003	15.02 vs. 12.10 (+19.4 %) *p* = 1.3354E-05	12.74 vs. 12.10 (+5.0 %) *p* = 0.2232
microRNA-99a	6.23 vs. 5.61 (+10.0 %) *p* = 0.0406	6.23 vs. 5.09 (+18.4 %) *p* = 0.0004	5.61 vs. 5.09 (+9.3 %) *p* = 0.0370
microRNA-100	6.91 vs. 5.18 (+25.0 %) *p* = 4.0492E-08	6.91 vs. 4.69 (+32.2 %) *p* = 2.9936E-11	5.18 vs. 4.69 (+9.5 %) *p* = 0.0275
microRNA-125b	3.34 vs. 2.57 (+22.9 %) *p* = 0.0001	3.34 vs. 2.31 (+30.8 %) *p* = 0.0001	2.57 vs. 2.31 (+10.2 %) *p* = 0.1968
microRNA-138	13.08 vs. 12.19 (+6.8 %) *p* = 0.0301	13.08 vs. 11.95 (+8.7 %) *p* = 0.0082	12.19 vs. 11.95 (+2.0 %) *p* = 0.4705
microRNA-147b	14.57 vs. 13.44 (+7.8 %) *p* = 0.0028	14.57 vs. 13.49 (+7.4 %) *p* = 0.0105	13.44 vs. 13.49 (−0.4 %) *p* = 0.8662
microRNA-148a	0.69 vs. 5.03 (−622.1 %) *p* = 3.9814E-09	0.69 vs. 5.29 (−660.5 %) *p* = 2.1259E-12	5.03 vs. 5.29 (−5.3 %) *p* = 0.5821
microRNA-210	9.17 vs. 8.26 (+9.9 %) *p* = 0.0044	9.17 vs. 7.56 (+17.6 %) *p* = 4.9353E-05	8.26 vs. 7.56 (+8.5 %) *p* = 0.0208
microRNA-376a	16.39 vs. 15.82 (+3.5 %) *p* = 0.1332	16.39 vs. 15.28 (+6.8 %) *p* = 0.0026	15.82 vs. 15.28 (+3.4 %) *p* = 0.0849

Benign, noninflammatory pancreatic specimens of patients with pancreatic cystadenoma or papillary adenoma served as controls. All data are given as median ΔCt level expression differences in percent. *p* < 0.05 indicates significance

Expression of in vitro dysregulated microRNA-376a, microRNA-330-3p, microRNA-330-5p, microRNA-378d, microRNA-422a, microRNA-455-3p, and microRNA-486-5p did not differ between PDAC and control.

**Fig. 3:**
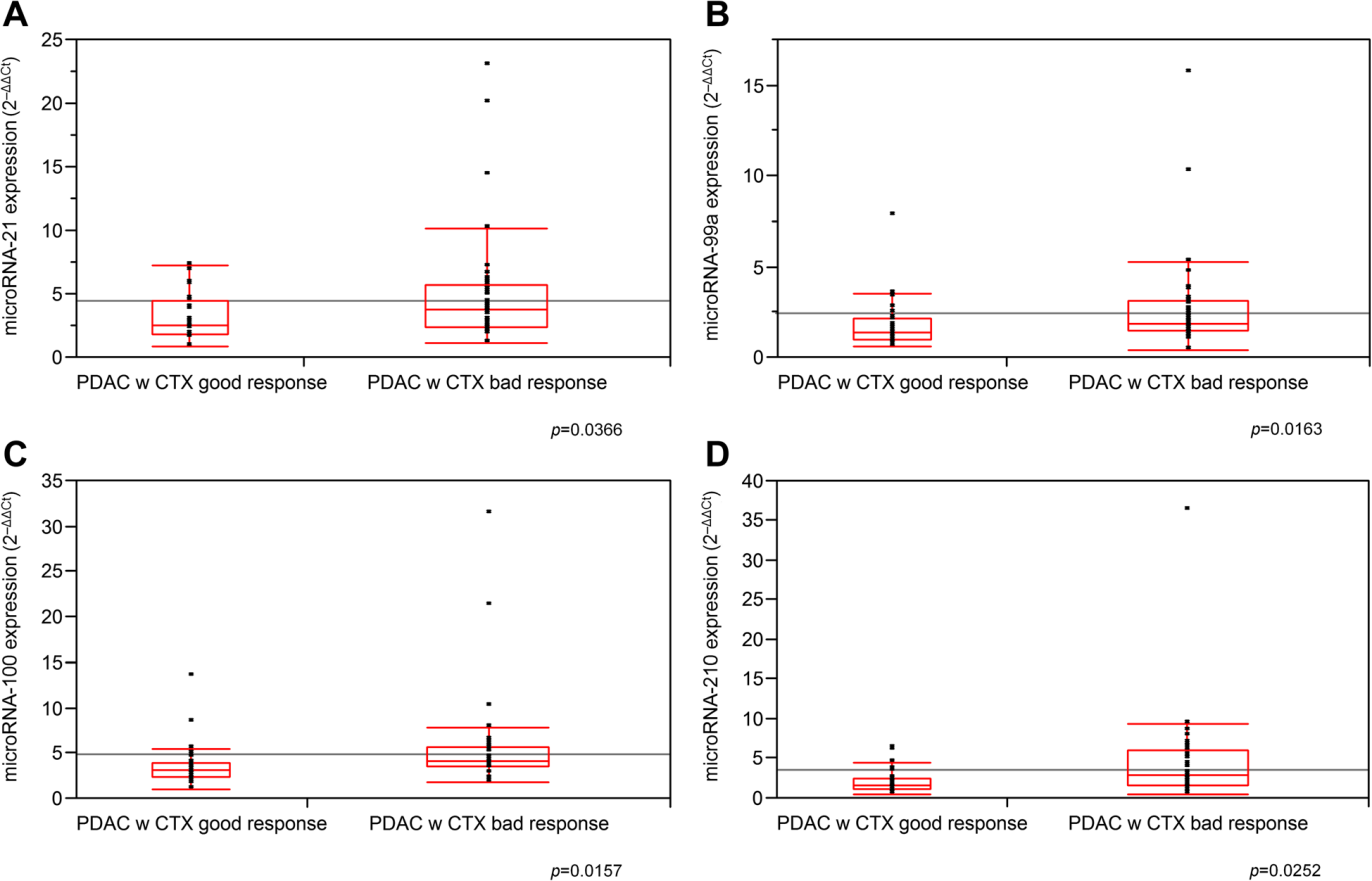
The 2^-ΔΔCt^ expression level of microRNA-21 (**a**), microRNA-99a (**b**), microRNA-100 (**c**), and microRNA-210 (**d**) in PDAC UICC stage II with good and bad response. *p* < 0.05 indicates significance

After normalization to benign noninflammatory controls (*n* = 13) by the ΔΔCt method, poor adjuvant gemcitabine mono-chemotherapy response was significantly related to overexpression of microRNA-21 (*p* = 0.0366), microRNA-99a (*p* = 0.0163), microRNA-100 (*p* = 0.0157), and microRNA-210 (*p* = 0.0252) (Fig. 3).

**Fig. 4:**
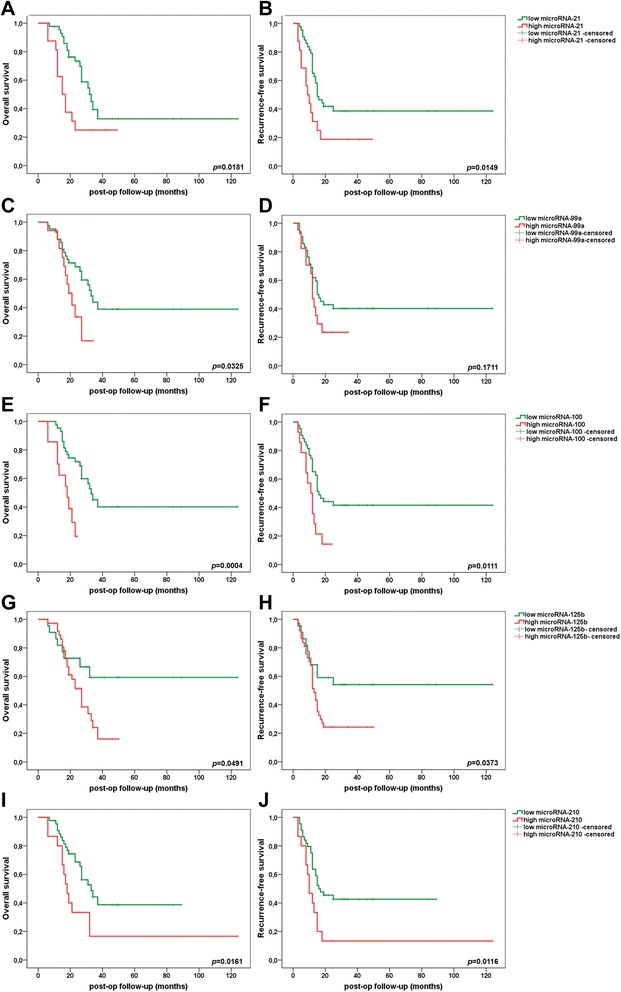
Prognostic impact of microRNA-21 (**a**, **b**), microRNA-99a (**c**, **d**), microRNA-100 (**e**, **f**), microRNA-125b (**g**, **h**), and microRNA-210 (**i**, **j**) on overall survival (*right column*) and recurrence-free survival (*left column*) in PDAC UICC stage II patients. *p* < 0.05 indicates significance

Kaplan-Meier survival analysis revealed significantly improved overall survival and recurrence-free survival rates in PDAC patients with low expression of microRNA-21 (cutoff 4.7; *p* = 0.0181; *p* = 0.0149), microRNA-99a (cutoff 2.5; *p* = 0.0325; *p* = 0.1711), microRNA-100 (cutoff 5.0; *p* = 0.0004; *p* = 0.0111), microRNA-125b (cutoff 1.6; *p* = 0.0491; *p* = 0.0373), and microRNA-210 (cutoff 4.6; *p* = 0.0161; *p* = 0.0116) in the adjuvant setting (Fig. 4). Univariate Cox regression overall and recurrence-free survival analyses identified microRNA-21 (*p* = 0.0231; *p* = 0.0211), microRNA-99a (*p* = 0.0393; *p* = 0.1864), microRNA-100 (*p* = 0.0013; *p* = 0.0163), microRNA-125b (*p* = 0.0578; *p* = 0.0472), and microRNA-210 (*p* = 0.0211; *p* = 0.0168) as unfavorable prognostic factors in resected and adjuvant-treated PDAC UICC stage II patients (Table 5). These microRNAs did not correlate significantly with standard clinicopathologic parameters or immunohistological markers (data not shown).

### Classic immunohistological PDAC chemoresistance und survival markers

**Fig. 5:**
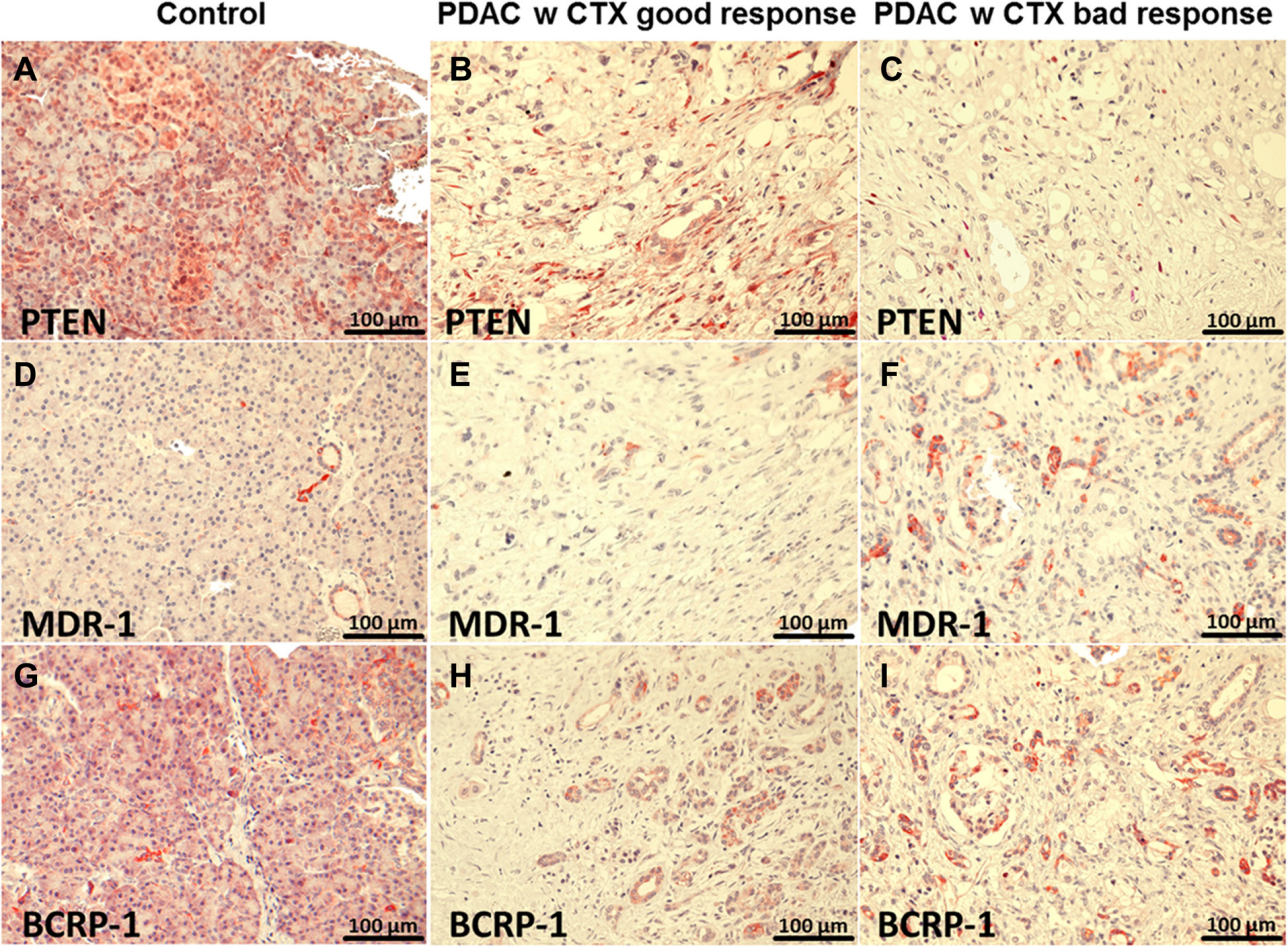
Immunohistochemical staining of PTEN, MDR-1, und BCRP-1 in benigne pancreas (**a**, **d**, **g**), PDAC UICC stage II with good (**b**, **e**, **h**) and poor chemotherapy response (**c**, **f**, **i**)

Corresponding formalin-fixed paraffin-embedded (FFPE) sections of our study groups with 91 patients with PDAC UICC stage II and 13 patients with pancreatic cystadenoma or papillary adenoma (total *n* = 104) were evaluated immunohistochemically by tissue microarray for the expression of the tumor suppressor PTEN, the gemcitabine metabolism regulators DCK and HuR, and the ATP transporters MDR-1, MRP-1, and BCRP-1, as well as the tumor angiogenesis marker vascular epithelial growth factor (VEGF)-1. MDR-1 (*p* = 0.0426) and BCRP-1 (*p* = 0.0378) overexpression as well as PTEN (*p* = 0.0394) underexpression correlated significantly with poor chemotherapy response in PDAC patients. VEGF-1 was significantly downregulated in PDAC tissue vs. benign controls (*p* < 0.0001) (Fig. 5, Table 4).

**Table 4:** Immunohistochemical staining density of different study groups

Antibody	Control (I)	PDAC w CTX		PDAC w/o CTX	*p*
		Good response (II)	Bad response (III)		
PTEN					
+4	3	1	0	0	I vs. II: 0.0901
+3	6	7	6	0	I vs. III: 0.0003
+2	4	14	16	12	II vs. III: 0.0394
+1	0	3	17	15	
DCK					
+4	7	10	6	1	I vs. II: 0.7549
+3	4	8	13	16	I vs. III: 0.0287
+2	2	6	15	9	II vs. III: 0.1147
+1	0	1	5	1	
HuR					
+4	0	0	0	0	I vs. II: 0.0687
+3	5	2	3	1	I vs. III: 0.0137
+2	5	13	13	8	II vs. III: 0.3054
+1	3	10	23	18	
MDR-1					
+4	0	0	0	0	I vs. II: 0.7427
+3	0	1	7	1	I vs. III: 0.1068
+2	2	3	11	5	II vs. III: 0.0426
+1	11	21	21	21	
MRP-1					
+4	9	1	4	1	I vs. II: 0.00001
+3	4	3	12	3	I vs. III: 0.0001
+2	0	6	11	10	II vs. III: 0.1005
+1	0	15	12	13	
BCRP-1					
+4	2	0	0	0	I vs. II: 0.00003
+3	8	1	7	2	I vs. III: 0.0005
+2	3	12	24	19	II vs. III: 0.0378
+1	0	12	8	6	
VEGF-1					
+4	10	0	1	0	I vs. II: <0.0001
+3	3	4	6	2	I vs. III: <0.0001
+2	0	14	23	12	II vs. III: 0.8456
+1	0	7	9	13	

*p* < 0.05 indicates significance

**Fig. 6:**
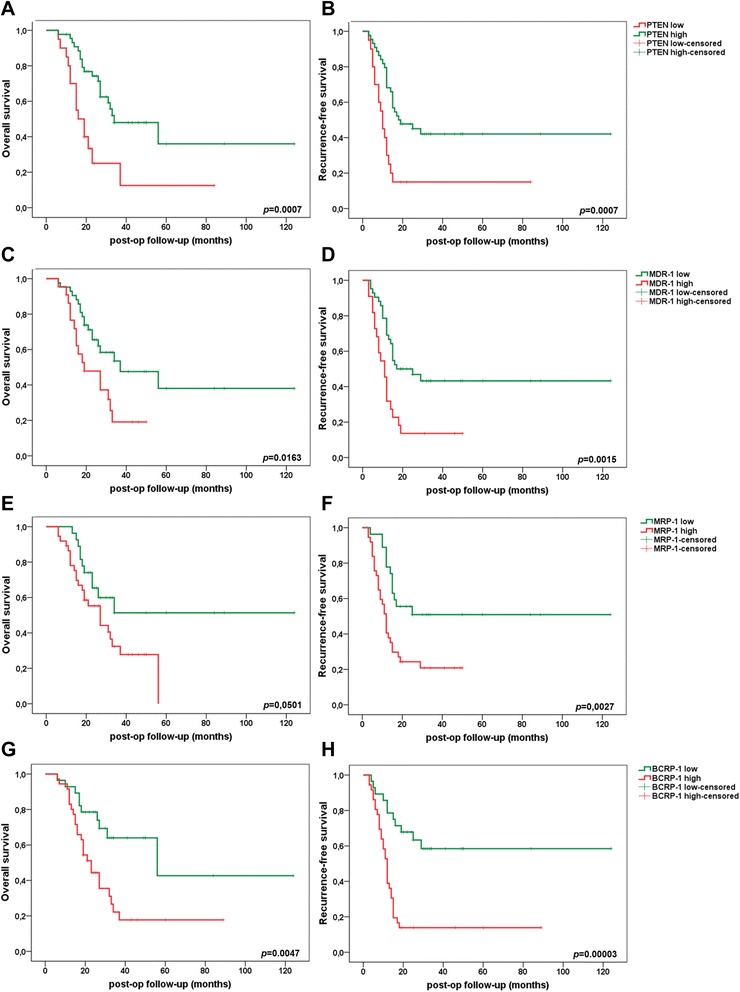
Prognostic impact of PTEN (**a**, **b**), MDR-1 (**c**, **d**), MRP-1 (**e**, **f**), and BCRP-1 (**g**, **h**) on overall survival (*right column*) and recurrence-free survival (*left column*) in PDAC UICC stage II patients. *p* < 0.05 indicates significance

Kaplan-Meier survival analysis revealed significantly improved overall survival and recurrence-free survival rates in PDAC patients with high expression of PTEN (*p* = 0.0007 each) and low expression of MDR-1 (*p* = 0.0163; *p* = 0.0015) and BCRP-1 (*p* = 0.0047; *p* = 0.00003) in the adjuvant setting (Fig. 6). Univariate Cox regression overall and recurrence-free survival analyses identified PTEN (*p* = 0.0014; *p* = 0.0015) and DCK (*p* = 0.0939; *p* = 0.0345) as favorable prognostic factors as well as MDR-1 (*p* = 0.0208; *p* = 0.0028), BCRP-1 (*p* = 0.0074; *p* = 0.0001), and MRP-1 (*p* = 0.0576; *p* = 0.0049) as unfavorable prognostic factors in resected and adjuvant-treated PDAC UICC stage II patients (Table 5).

**Table 5:** Univariate Cox regression overall and recurrence-free survival analysis

Variable	Subset	Overall survival		Recurrence-free survival	
		Hazard ratio (95 % CI)	*p* value	Hazard ratio (95 % CI)	*p* value
PTEN	low/high	3.095 (1.5502–6.1805)	0.0014	2.7944 (1.4826–5.2669)	0.0015
DCK	low/high	0.5536 (0.2772–1.1057)	0.0939	0.5178 (0.2813–0.9532)	0.0345
HuR	low/high	0.6799 (0.3448–1.3409)	0.2656	0.6569 (0.3559–1.2125)	0.1789
MDR-1	low/high	2.2213 (1.1291–4.3698)	0.0208	2.5516 (1.3799–4.7179)	0.0028
MRP-1	low/high	2.0104 (0.9778–4.1331)	0.0576	2.5762 (1.3334–4.9773)	0.0049
BCRP-1	low/high	2.7438 (1.3117–5.7395)	0.0074	3.9656 (1.9563–8.0385)	0.0001
VEGF-1	low/high	1.5977 (0.6059–4.2123)	0.3435	1.3889 (0.6157–3.1331)	0.4286
N	N0/N1	1.2670 (0.6269–2.5605)	0.5096	1.16965 (0.6213–2.2018)	0.6273
G	G3/G1-2	0.9045 (0.4425–1.8491)	0.7833	0.7767 (0.4036–1.4946)	0.4492
L	L0/L1	1.2793 (0.6478–2.5264)	0.4781	1.1996 (0.6541–2.1998)	0.5565
V	V0/V1	1.4841 (0.6944–3.1716)	0.3083	1.7039 (0.8545–3.3975)	0.1301
Pn	Pn0/Pn1	1.3512 (0.6629–2.7538)	0.4073	1.2389 (0.6426–2.3889)	0.5224
R	0/1	1.3996 (0.6839–2.8644)	0.3576	1.8921 (0.9923–3.6081)	0.0528
gender	f/m	0.9404 (0.4784–1.8487)	0.8586	1.0039 (0.5444–1.8513)	0.9899
age	>65/≤65	0.7334 (0.3747–1.4354)	0.3655	0.7598 (0.4142–1.3938)	0.3749
operation	DP/PD	1.1089 (0.5034–2.4431)	0.7974	1.2319 (0.5889–2.5774)	0.5796
CTX response	good/bad	23.3621 (5.5057–99.1321)	0.00002	159.7685 (11.1902–2281.0912)	0.0002
microRNA-21	low/high	(2.3127) (1.1218–4.7675)	0.0231	2.2006 (1.1256–4.3022)	0.0211
microRNA-31*	low/high	1.4769 (0.7365–2.9614)	0.2719	1.7738 (0.9362–3.3606)	0.0788
microRNA-99	low/high	2.2291 (1.0399–4.7781)	0.0393	1.5775 (0.8023–3.1019)	0.1864
microRNA-100	low/high	4.0097 (1.7189–9.3536)	0.0013	2.3653 (1.1714–4.7762)	0.0163
microRNA-125b	low/high	2.1879 (0.9745–4.9127)	0.0578	2.0935 (1.0093–43426)	0.0472
microRNA-138	low/high	1.4770 (0.6583–3.3141)	0.3442	1.3268 (0.6556–2.6854)	0.4318
microRNA-147b	low/high	0.8217 (0.4094–1.6489)	0.5805	0.7387 (0.3891–1.4022)	0.3543
microRNA-148a	low/high	0.9135 (0.4533–1.8411)	0.8003	1.1714 (0.6249–2.1957)	0.6217
microRNA-210	low/high	2.4021 (1.1408–5.0578)	0.0211	2.2881 (1.1611–4.5091)	0.0168
microRNA-376a	low/high	1.9456 (0.9518–3.9771)	0.0681	1.7029 (0.8906–3.2563)	0.1075

Indication of hazard ratio and 95 % confidence interval (CI). *p* < 0.05 indicates significance

### Identification of independent prognostic factors by multivariate analysis

Multivariate Cox regression overall and recurrence-free survival analyses identified microRNA-21 (*p* = 0.0064; *p* = 0.0338), microRNA-100 (*p* = 0.0045; *p* = 0.1683), and MDR-1 (*p* = 0.0003; *p* = 0.0021) as unfavorable prognostic factors while PTEN (*p* = 0.1008; *p* = 0.0211) was a favorable prognostic factor in resected and adjuvantly treated PDAC UICC stage II patients (Table 6). As combinations of microRNAs are likely to provide more accurate prognostic information, the most accurate subset of variables (microRNA-21, -31*, -99, -100, -125b, -138, -147b, -148a, -210, and -376a) was identified using multivariate survival analysis with a forward stepwise variable selection procedure based on the likelihood ratio. Co-expression of microRNA-21 and microRNA-100 as remaining independent prognostic factors results in a hazard ratio of 2.620 (95 % CI 1,251-5,487) and 3.543 (95 % CI 1.504-8.345), respectively.

**Table 6:** Multivariate Cox regression overall and recurrence-free survival analysis

Variable	Subset	Overall survival		Recurrence-free survival	
		Hazard ratio (95 % CI)	*p* value	Hazard ratio (95 % CI)	*p* value
PTEN	low/high	0.6076 (0.3351–1.1017)	0.1008	0.5431 (0.3232–0.9125)	0.0211
MDR-1	low/high	3.0434 (1.6602–5.5790)	0.0003	2.1309 (1.3164–3.4494)	0.0021
BCRP-1	low/high	0.7729 (0.3924–1.5225)	0.4565	1.3209 (0.7463–2.3378)	0.3394
microRNA-21	low/high	3.0649 (1.3709–6.8522)	0.0064	2.2521 (1.0640–4.7666)	0.0338
microRNA-100	low/high	4.8450 (1.6306–14.3961)	0.0045	1.8714 (0.7673–4.5643)	0.1683
microRNA-210	low/high	1.2116 (0.4849–3.0270)	0.6812	1.7623 (0.7634–4.0681)	0.1843

Indication of hazard ratio and 95 % confidence interval (CI). *p* < 0.05 indicates significance

## Discussion

As pancreatic cancer surgery and perioperative treatment have significantly improved over the past decades, today, resistance to chemotherapy and chemotoxicity remain as important aspects of treatment failure in resectable PDAC patients.

The high mortality of locally advanced and metastatic PDAC could be reduced by increasing susceptibility to adjuvant gemcitabine chemotherapy. Currently, no tissue biomarkers are available to guide therapeutic strategies or predict prognosis and chemotherapy response in PDAC patients.

Several prognostic clinical factors of survival have been identified in PDAC patients [12, 13]. In accordance with the literature*,* low tumor grading and adjuvant gemcitabine chemotherapy were correlated to improved survival in PDAC UICC stage II patients in this study. However, with the exception of adjuvant gemcitabine treatment, none of the investigated clinicopathological parameters were independent prognostic markers in the adjuvant setting. Instead, we identified microRNA-100 and microRNA-21 with its targets PTEN and MDR-1 as independent prognostic survival and chemotherapy response markers in PDAC UICC stage II patients.

The oncogenic role of microRNA-21 in PDAC patients and its association with poor survival and induction of chemoresistance to gemcitabine is already well known [9, 14]. Increased microRNA-21 expression has been shown to promote chemoresistance through repression of the tumor suppressor PTEN and decreased expression of programmed cell death 4 (PDCD4). Interestingly, decrease in PDCD4 levels in turn increases the expression of apoptosis inhibitors and the multidrug resistance protein MDR-1 [15–17]. In this study, high expression of PTEN and low expression of MDR-1 was accompanied by significantly improved overall and recurrence-free survival as well as chemotherapy response. However, the clinical significance of MDR-1 expression in human PDAC is controversial. Some research groups failed to demonstrate MDR-1 expression in PDAC tissues, whereas other studies showed a common and high MDR-1 expression potentially contributing to the chemoresistant biology of PDAC [5, 18–21]. VEGF has been reported as a tumor angiogenesis marker in PDAC, positively correlating with microRNA-21 expression [9, 22]. However, the significance of VEGF in tumor neoangiogenesis and progression in PDAC is still unclear. Literature on the prognostic value of VEGF in PDAC shows conflicting findings, and phase II–III clinical trials did not reveal any benefit from combined gemcitabine anti-VEGF PDAC treatment [23]. Our data could not confirm a prognostic role of VEGF in PDAC.

We validated several microRNAs in clinical tumor samples that we had previously identified to be dysregulated in vitro [11]. The identified chemoresistance-associated microRNAs 99a, 100, 125b, and 210 are already known as potential oncogenes in PDAC. In smaller clinical studies, microRNA-210 has been reported to be overexpressed in PDAC patients and associated with a worse outcome [24]. microRNA-99a and microRNA-100, two members of the microRNA-99 family, were found to be overexpressed in PDAC tissue compared with normal pancreatic tissue and chronic pancreatitis [25]. Overexpression of microRNA-125b was demonstrated in prostate cancer, glioblastoma, and drug resistance in pediatric leukemia and breast cancer [26–29]. Interestingly, co-overexpression of miR-125b, miR-99a, and miR-100 was reported in different cancers suggests possible co-regulation of these miRs in chemoresistant PDAC as well [30, 31].

In addition to microRNA-21, microRNA-100 remained as a further independant prognostic survival and chemotherapy response marker in multivariate analysis. Further multi-center studies with a larger number of PDAC patients are required to reevaluate the impact of microRNA-99a, microRNA-125b, and microRNA-210 as independant survival predictors.

## Conclusions

We have identified a panel of microRNAs that could serve as survival and chemotherapy response markers in PDAC patients UICC stage II. In particular, microRNA-21 and microRNA-100 show promise as molecular markers and key regulators for targeted therapies in chemorefractive PDAC patients.

## Methods

### Patients and samples

A tissue bank and follow-up database are maintained prospectively by the Department of General and Visceral Surgery and the Comprehensive Cancer Center Muenster, University Hospital Muenster, Muenster, Germany. From these, 98 PDAC UICC stage II tumor specimens having undergone resection between 2003 and 2012 were retrieved. Benign, noninflammatory pancreatic specimens of 13 patients with pancreatic cystadenoma or papillary adenoma served as controls. Tissue samples were immediately fixed in 10 % buffered formalin and then processed into a paraffin-embedded block and stored at room temperature. Sections from each of the 111 specimens were examined by a pathologist and graded histologically. All cancerous specimens showed vital tumor tissue.

Ethical approval for postoperative tissue collection was obtained (Ethics committee, University Muenster, Az: 1IXHai v. 19.9.2001), and all patients provided informed written consent. All patients underwent radical resection and were assigned to duodenopancreatectomy or left pancreatic resection. Patients that received immunosuppression, neoadjuvant chemo-, or radiotherapy were excluded to avoid potential influences on microRNA expression. Perioperative clinical data, histopathological information, and follow-up data were collected for all patients using the clinical cancer registry. A poor chemotherapy response was defined as a tumor recurrence or a tumor-related death of ≤18 months (cutoff) postoperatively. The cutoff of 18 months was selected due to the median survival of 13–18 months of PDAC patients with curative treatment intent (median split method) [32]. The primary end points of this study were recurrence-free survival and overall survival.

### Tumor macrodissection and RNA purification

For each histological sample, a representative hematoxylin and eosin (H&E)-stained section was reviewed by a trained and experienced pathologist. Tissue samples included in this study had >60 % viable cells and <20 % necrosis. Tumor and stromal tissue areas were selectively distinguished with a permanent marker by the pathologist to guide macrodissection. Macrodissection of samples with adenoma was carried out to create benign controls using sample material distant from adenoma. Twenty-micrometer sections from each FFPE tissue sample were cut with a microtome and processed immediately. After melting the paraffin block at 65 °C for 1 h to liberate the tissue from the surrounding paraffin, separation of the tumor tissue from the stromal area with a scalpel was carried out. Cut sections were placed in 1.5 ml tubes for RNA extraction.

Total RNA enriched in microRNAs fraction was purified through robotic workstation (QIAcube, Qiagen) by using miRNeasy FFPE Kit isolation system following manufacturer’s protocols (Qiagen). RNA concentration and purity were assessed by Agilent 2100 Bioanalyzer and RNA 6000 Nano/Pico LabChip (Agilent Tech., Boeblingen, Germany). Samples with RIN >7 were considered intact and used for analysis. RNA was stored at −80 °C.

### Selection of microRNAs

Sixteen significantly deregulated microRNAs in two human PDAC cell lines (MIA-PaCa-2 and PANC-1) with acquired gemcitabine chemoresistance had been previously identified. Our prior in vitro study with Affymetrix GeneChip miRNA microarray (Affymetrix UK Ltd., High Wycombe, UK) analysis of 1733 human microRNAs and validation by qRT-PCR showed microRNA-21, microRNA-99a, microRNA-100, microRNA-125b, microRNA-138, microRNA-147b, microRNA-148a, microRNA-210, microRNA-376a, and microRNA-455-3p to be significantly upregulated, whereas microRNA-31-star, microRNA-330-3p, microRNA-330-5p, microRNA-378d, microRNA-422a, and microRNA-486-5p were significantly downregulated. Bioinformatic analysis and Western blot validation suggested involvement of these microRNAs in pathways controlling cell death and cycle [11].

MicroRNA expression data were normalized to expression levels of the four housekeeping genes RNU1A, RNU6-2, SNORD68, and SNORD96A selected from a total of ten tested housekeeping genes.

### Quantification of microRNAs

qRT-PCR was performed using the miScript PCR system (Qiagen). Total RNA samples (1 μg) were reverse transcribed to complementary DNA (cDNA) using miScript II Reverse Transcription Kit (Qiagen). For each sample, 2 μl of the generated cDNA was mixed with 10 μl 2× QuantiTect SYBR, 2 μl 10× miScript Universal Primer, 2 μl gene specific 10× miScript Primer Assay (for five different miRNAs), and 1 μl nuclease free water. All samples were analyzed in triplicate using Bio-Rad CFX384TM RT PCR cycler (Bio-Rad Laboratories, Munich, Germany) with miScript SYBR Green PCR Kit (Qiagen). No template and minus RT controls were run in parallel. The cycling program involved an initial incubation step of 15 min at 95 °C to activate HotStarTaq DNA Polymerase, followed by 40 cycles of denaturation at 94 °C for 15 s, annealing at 55 °C for 30 s and elongation at 70 °C for 30 s. Quantitative miR analysis was performed using CFX Manager Software v2.1 (Bio-Rad Laboratories). Expression of the selected microRNAs was analyzed quantitatively relative to the four housekeeping genes (hg) by the 2^–ΔΔCT^ method using the equation: relative quantity *R* = 2^–ΔΔCt^, where ΔΔCt = (Ct microRNA − Ct hg) cancer − (Ct microRNA − CtT hg) benign and Ct is the cycle threshold [33].

### Tissue array immunohistochemistry

For the tissue microarray (TMA), corresponding H&E-stained sections from each FFPE block were used to define and to mark representative tumor areas by the pathologist, two to four random, representative 2-mm tissue cores were punched from the designated area using a biopsy needle and inserted in a grid pattern into a recipient paraffin block using a tissue arrayer [34]. The final TMA contained 44 tissue cores processed in duplicate. Four-micrometer-thick sections were cut from each completed TMA array block and transferred to silanized glass slides. Sections from these arrays were then stained with the panel of antibodies: phosphatase and tensin homolog (PTEN) (monoclonal antibody, 1:200, clone NCH-38, Dako, Hamburg, Germany), deoxycytidine kinase (DCK) (monoclonal antibody, 1:5000, clone V9, Dako, Hamburg, Germany), HuR, MRP-1, MDR-1, BCRP-1, VEGF-1, and lymphatic endothelial marker D2-40. These antibodies were chosen based on their use as markers of tumor suppression, gemcitabine transport, and metabolism [5, 6, 8, 9, 35–37].

All immunohistochemical TMA studies were performed by the avidin-biotin-peroxidase complex method of Hsu et al. [38] For immunostaining, the Dako Autostainer Plus S3400 and the Dako Real Detection System (Alkaline Phosphatase/RED, Rabbit/Mouse, K5005) were used according to the manufacturer’s instructions (Dako, Hamburg, Germany). Briefly, after dewaxing and epitope recovery for 35 min at 98 °C, endogenous peroxidase activity was quenched by Dako Real Peroxidase-Blocking Solution for 10 min at room temperature. Afterwards, slides were incubated with primary antibodies and diluted in Dako Real antibody diluent, for 25 min. This step was followed by incubation with Dako Real Link, biotinylated secondary antibodies (20 min), incubation with Dako Real Streptavidin Alkaline Phosphatase (20 min), and visualization by RED chromogen (2 × 8 min) using the Dako autostaining system with appropriate controls for each run. Finally, slides were counterstained with Mayer’s hematoxylin and dehydrated in graded alcohols. Positive controls were included in all experiments using the same experimental conditions. A negative control was prepared by the same staining procedure without primary antibodies.

Immunohistochemical staining was evaluated semi-quantitatively, according to the percentage of cells showing specific immunoreactivity. Two independent investigators evaluated, in a blinded manner, antibody staining using light microscopy (Eclipse E1000M and NIS-Elements D3.1 imaging software, Nikon, Tokyo, Japan) and a scoring system with a scale ranging from 0 to 4+ [5]. Disagreements were resolved by joint review. For each case, the core with the highest percentage of tumor cell staining was used for analysis. Grade 0 was defined as complete absence of staining. Grade 1+ was defined as a low positive staining up to 25 %. Grade 2+ was defined as a moderate positive staining density of ≥25 but <50 %. A positive high staining density ≥50 % but <75 % of cells was defined as grade 3+. Finally, a positive staining density ≥75 % was assessed as grade 4+. Staining intensity was scored as weak, moderate, or strong.

### Statistical analysis

Statistical analysis was performed with the SPSS® Statistics Version 22 (IBM Corp. Armonk, NY) for Windows®. Data were expressed as medians of mean normalized expression with standard deviation (SD). Statistical significance was determined by using the two-tailed Student’s *t*test to compare two data sets.

Box-and-whisker plots demonstrating the median (middle quartile) were used to show normalized microRNA expression data (2^-ΔΔCt^) in the different therapy response groups. The middle half of a data set falls within the interquartile range represented by the width of the box. The whiskers indicate variability outside the upper and lower quartiles. The grand mean representing the overall mean of all data combined is displayed as a horizontal line across the entire graph. Comparison between gene expression and categorical variables was conducted using the nonparametric Mann-Whitney *U* test or the Kruskal-Wallis test to test more than two groups. To test the correlation between the clinicopathological data and the expression of the microRNAs, we used the Fisher two-tailed exact test and whenever appropriate the *χ*^2^ test. All of the variables were dichotomized. For analysis of follow-up data, life table curves were calculated using the Kaplan-Meier method. Log-rank test was used for comparison of relapse-free survival rates and survival rates between the groups with high and low microRNA or TAM marker expression in PDAC tissue compared to benign controls. For the identification of the best cutoff value for a supposed prognostic variable the log-rank test was performed for all observed values within the entire data set according to LeBlanc et al. [39] The primary end points were disease-specific survival or relapse-free survival, as measured from the date of surgery to the time of the last follow-up or cancer-related death or tumor relapse, respectively. Data of patients who were still alive and without evidence of tumor relapse at the end of the study were censored. A Cox proportional-hazards regression model was used to estimate hazard ratios and 95 % confidence intervals (CIs) and to perform multivariate survival analysis using a forward stepwise variable selection procedure based on the likelihood ratio. Variables with significant *p* values in the univariate analysis were included in the multivariate analysis. *p* value <0.05 was considered to be statistically significant. Housekeeping gene selection was performed by geNorm software (Biogazelle NV, Zwijnaarde, Belgium), a popular algorithm to determine the most stable reference (housekeeping) genes from a set of tested candidate reference genes (*n* = 10). The gene expression stability measure (M) for a reference gene is calculated by geNorm as the average pairwise variation for that gene with all other tested reference genes. Housekeeping genes with an average expression stability M ≤ 0.5 were considered to have a high target reference stability [40]. By this way, we identified four housekeeping genes for accurate microRNA qRT-PCR data normalization.

## Abbreviations

ABC: ATP binding cassette
BCRP: breast cancer resistance protein
DCK: deoxycytidine kinase
FFPE: formalin-fixed paraffin-embedded
H&E: hematoxylin and eosin
HuR: Hu antigen R
MDR: multidrug resistance
MRP: multidrug resistance protein
PDAC: pancreatic ductal adenocarcinoma
PTEN: phosphatase and tensin homolog
TMA: tissue microarray
UICC: Union Internationale Contre le Cancer
VEGF: vascular epithelial growth factor

## References

[1] Siegel RL, Miller KD, Jemal A (2015). Cancer statistics, 2015. CA Cancer J Clin.

[2] Vincent A, Herman J, Schulick R, Hruban RH, Goggins M (2011). Pancreatic cancer. Lancet.

[3] Von Hoff DD, Ramanathan RK, Borad MJ, Laheru DA, Smith LS, Wood TE (2011). Gemcitabine plus nab-paclitaxel is an active regimen in patients with advanced pancreatic cancer: a phase I/II trial. J Clin Oncol.

[4] Conroy T, Desseigne F, Ychou M, Bouche O, Guimbaud R, Becouarn Y (2011). FOLFIRINOX versus gemcitabine for metastatic pancreatic cancer. N Engl J Med.

[5] O’Driscoll L, Walsh N, Larkin A, Ballot J, Ooi WS, Gullo G (2007). MDR1/P-glycoprotein and MRP-1 drug efflux pumps in pancreatic carcinoma. Anticancer Res.

[6] Marechal R, Mackey JR, Lai R, Demetter P, Peeters M, Polus M (2010). Deoxycitidine kinase is associated with prolonged survival after adjuvant gemcitabine for resected pancreatic adenocarcinoma. Cancer.

[7] Costantino CL, Witkiewicz AK, Kuwano Y, Cozzitorto JA, Kennedy EP, Dasgupta A (2009). The role of HuR in gemcitabine efficacy in pancreatic cancer: HuR up-regulates the expression of the gemcitabine metabolizing enzyme deoxycytidine kinase. Cancer Res.

[8] Richards NG, Rittenhouse DW, Freydin B, Cozzitorto JA, Grenda D, Rui H (2010). HuR status is a powerful marker for prognosis and response to gemcitabine-based chemotherapy for resected pancreatic ductal adenocarcinoma patients. Ann Surg.

[9] Giovannetti E, Funel N, Peters GJ, Del Chiaro M, Erozenci LA, Vasile E (2010). MicroRNA-21 in pancreatic cancer: correlation with clinical outcome and pharmacologic aspects underlying its role in the modulation of gemcitabine activity. Cancer Res.

[10] Dhayat S, Mardin WA, Mees ST, Haier J (2011). Epigenetic markers for chemosensitivity and chemoresistance in pancreatic cancer—a review. Int J Cancer.

[11] Dhayat SA, Mardin WA, Seggewiß J, Ströse AJ, Matuszcak C, Hummel R (2015). MicroRNA profiling implies new markers of gemcitabine chemoresistance in mutant p53 pancreatic ductal adenocarcinoma. PLoS One.

[12] Luttges J, Schemm S, Vogel I, Hedderich J, Kremer B, Kloppel G (2000). The grade of pancreatic ductal carcinoma is an independent prognostic factor and is superior to the immunohistochemical assessment of proliferation. J Pathol.

[13] Lim JE, Chien MW, Earle CC (2003). Prognostic factors following curative resection for pancreatic adenocarcinoma: a population-based, linked database analysis of 396 patients. Ann Surg.

[14] Hwang JH, Voortman J, Giovannetti E, Steinberg SM, Leon LG, Kim YT (2010). Identification of microRNA-21 as a biomarker for chemoresistance and clinical outcome following adjuvant therapy in resectable pancreatic cancer. PLoS One.

[15] Bourguignon LY, Spevak CC, Wong G, Xia W, Gilad E (2009). Hyaluronan-CD44 interaction with protein kinase C(epsilon) promotes oncogenic signaling by the stem cell marker Nanog and the production of microRNA-21, leading to down-regulation of the tumor suppressor protein PDCD4, anti-apoptosis, and chemotherapy resistance in breast tumor cells. J Biol Chem.

[16] Allen KE, Weiss GJ (2010). Resistance may not be futile: microRNA biomarkers for chemoresistance and potential therapeutics. Mol Cancer Ther.

[17] Gisel A, Valvano M, El Idrissi IG, Nardulli P, Azzariti A, Carrieri A (2014). miRNAs for the detection of multidrug resistance: overview and perspectives. Molecules.

[18] Sugawara I, Kataoka I, Morishita Y, Hamada H, Tsuruo T, Itoyama S (1988). Tissue distribution of P-glycoprotein encoded by a multidrug-resistant gene as revealed by a monoclonal antibody, MRK 16. Cancer Res.

[19] Benard J, Bourhis J, Riou G (1990). Clinical significance of multiple drug resistance in human cancers. Anticancer Res.

[20] Chen M, Xue X, Wang F, An Y, Tang D, Xu Y (2012). Expression and promoter methylation analysis of ATP-binding cassette genes in pancreatic cancer. Oncol Rep.

[21] Suwa H, Ohshio G, Arao S, Imamura T, Yamaki K, Manabe T (1996). Immunohistochemical localization of P-glycoprotein and expression of the multidrug resistance-1 gene in human pancreatic cancer: relevance to indicator of better prognosis. Jpn J Cancer Res.

[22] Moriyama T, Ohuchida K, Mizumoto K, Yu J, Sato N, Nabae T (2009). MicroRNA-21 modulates biological functions of pancreatic cancer cells including their proliferation, invasion, and chemoresistance. Mol Cancer Ther.

[23] O’Reilly EM (2009). Pancreatic adenocarcinoma: new strategies for success. Gastrointest Cancer Res.

[24] Ho AS, Huang X, Cao H, Christman-Skieller C, Bennewith K, Le QT (2010). Circulating miR-210 as a novel hypoxia marker in pancreatic cancer. Transl Oncol.

[25] Bloomston M, Frankel WL, Petrocca F, Volinia S, Alder H, Hagan JP (2007). MicroRNA expression patterns to differentiate pancreatic adenocarcinoma from normal pancreas and chronic pancreatitis. Jama.

[26] Shi XB, Xue L, Ma AH, Tepper CG, Kung HJ, White RW (2011). miR-125b promotes growth of prostate cancer xenograft tumor through targeting pro-apoptotic genes. Prostate.

[27] Wu N, Lin X, Zhao X, Zheng L, Xiao L, Liu J (2013). MiR-125b acts as an oncogene in glioblastoma cells and inhibits cell apoptosis through p53 and p38MAPK-independent pathways. Br J Cancer.

[28] Zhou M, Liu Z, Zhao Y, Ding Y, Liu H, Xi Y (2010). MicroRNA-125b confers the resistance of breast cancer cells to paclitaxel through suppression of pro-apoptotic Bcl-2 antagonist killer 1 (Bak1) expression. J Biol Chem.

[29] Emmrich S, Rasche M, Schoning J, Reimer C, Keihani S, Maroz A (2014). miR-99a/100 ~125b tricistrons regulate hematopoietic stem and progenitor cell homeostasis by shifting the balance between TGFbeta and Wnt signaling. Genes Dev.

[30] Song T, Xia W, Shao N, Zhang X, Wang C, Wu Y (2010). Differential miRNA expression profiles in bladder urothelial carcinomas. Asian Pac J Cancer Prev.

[31] Nam EJ, Yoon H, Kim SW, Kim H, Kim YT, Kim JH (2008). MicroRNA expression profiles in serous ovarian carcinoma. Clin Cancer Res.

[32] Wilkowski R, Wolf M, Heinemann V (2008). Primary advanced unresectable pancreatic cancer. Recent Results Cancer Res.

[33] Livak KJ, Schmittgen TD (2001). Analysis of relative gene expression data using real-time quantitative PCR and the 2(−delta delta C(T)) method. Methods.

[34] Kallioniemi OP, Wagner U, Kononen J, Sauter G (2001). Tissue microarray technology for high-throughput molecular profiling of cancer. Hum Mol Genet.

[35] Nath S, Daneshvar K, Roy LD, Grover P, Kidiyoor A, Mosley L (2013). MUC1 induces drug resistance in pancreatic cancer cells via upregulation of multidrug resistance genes. Oncogenesis.

[36] Hamada S, Satoh K, Hirota M, Kanno A, Umino J, Ito H (2012). The homeobox gene MSX2 determines chemosensitivity of pancreatic cancer cells via the regulation of transporter gene ABCG2. J Cell Physiol.

[37] Georgiadou D, Sergentanis TN, Sakellariou S, Filippakis GM, Zagouri F, Vlachodimitropoulos D (2014). VEGF and Id-1 in pancreatic adenocarcinoma: prognostic significance and impact on angiogenesis. Eur J Surg Oncol.

[38] Hsu SM, Raine L, Fanger H (1981). A comparative study of the peroxidase-antiperoxidase method and an avidin-biotin complex method for studying polypeptide hormones with radioimmunoassay antibodies. Am J Clin Pathol.

[39] LeBlanc M, Jacobson J, Crowley J (2002). Partitioning and peeling for constructing prognostic groups. Stat Methods Med Res.

[40] Hellemans J, Mortier G, De Paepe A, Speleman F, Vandesompele J (2007). qBase relative quantification framework and software for management and automated analysis of real-time quantitative PCR data. Genome Biol.

